# A Quaternary Mechanism Enables the Complex Biological Functions of Octameric Human UDP-glucose Pyrophosphorylase, a Key Enzyme in Cell Metabolism

**DOI:** 10.1038/srep09618

**Published:** 2015-04-10

**Authors:** Jana Indra Führing, Johannes Thomas Cramer, Julia Schneider, Petra Baruch, Rita Gerardy-Schahn, Roman Fedorov

**Affiliations:** 1 Institute for Cellular Chemistry, Hannover Medical School, Carl-Neuberg-Str. 1, 30625 Hannover, Germany; 2 Research Division for Structural Analysis, Hannover Medical School, Carl-Neuberg-Str. 1, 30625 Hannover, Germany; 3 Institute for Biophysical Chemistry, Hannover Medical School, Carl-Neuberg-Str. 1, 30625 Hannover, Germany

## Abstract

In mammals, UDP-glucose pyrophosphorylase (UGP) is the only enzyme capable of activating glucose-1-phosphate (Glc-1-P) to UDP-glucose (UDP-Glc), a metabolite located at the intersection of virtually all metabolic pathways in the mammalian cell. Despite the essential role of its product, the molecular basis of UGP function is poorly understood. Here we report the crystal structure of human UGP in complex with its product UDP-Glc. Beyond providing first insight into the active site architecture, we describe the substrate binding mode and intermolecular interactions in the octameric enzyme that are crucial to its activity. Importantly, the quaternary mechanism identified for human UGP in this study may be common for oligomeric sugar-activating nucleotidyltransferases. Elucidating such mechanisms is essential for understanding nucleotide sugar metabolism and opens the perspective for the development of drugs that specifically inhibit simpler organized nucleotidyltransferases in pathogens.

Evolution has chosen glucose (Glc) as the central nutrient in almost all living systems. In mammals, Glc is essential for the long term supply of the central nervous system and the only substrate that can be metabolized by erythrocytes. Consequent to its pivotal nature, biological systems have evolved pathways to produce Glc from all major aliments^1^. Beyond its function as a nutrient, Glc is a key substrate in anabolic pathways, and processes like glycoprotein folding control, cellular detoxification and lactation rely on the availability of this sugar (Fig. 1). The use of Glc in all these pathways depends on its activation to UDP-glucose (UDP-Glc) in a reaction catalyzed by UDP-glucose pyrophosphorylase (UGP; EC 2.7.7.9) (Fig. 1). UGP follows an ordered sequential Bi Bi mechanism in both directions^2^ and uses Mg^2+^ as an essential cofactor^3^. With the exception of plants and certain protozoa, where a second enzyme with broader substrate specificity can form UDP-Glc^4^,^5^,^6^,^7^, UGP is the only enzyme capable of producing UDP-Glc from glucose-1-phosphate (Glc-1-P) and uridine triphosphate (UTP). Consistent with its vital role, no eukaryotic UGP loss-of-function mutants are known to occur naturally, and the only mammalian model system of impaired UGP function is a hamster fibroblast cell line in which a point mutation in the UGP gene causes a dramatic reduction of UDP-Glc levels^8^. In this cell line, an inactivation of glycogen synthase^9^, a hypersensitivity to toxins^10^ and increased cellular stress responses^11^ were observed. In *S. cerevisiae*, a UGP knock-down reduces cell wall stability and the knock-out is lethal^12^.

While no significant conservation exists between UGPs from pro- and eukaryotes^13^, the homology among eukaryotes is high even when comparing species as distant as mammals and plants^14^. The available crystal structures revealed eukaryotic UGPs to comprise a structurally conserved central catalytic domain with Rossmann-like α/β/α sandwich fold (reviewed in Kleczkowski *et al.*^15^), flanked by a flexible N-terminal domain with low conservation at primary sequence level and regulatory function in *S. cerevisiae*^16^, as well as a C-terminal β-helix domain^17^,^18^,^19^. In contrast to the primary, secondary and tertiary structures, the quaternary organizations of UGPs differ greatly. In diverse plants^19^,^20^,^21^ as well as the protozoan parasites *Leishmania major*^14^ and *Trypanosoma brucei*^22^, the active species of UGP was shown to be a monomer, while animal and fungal UGPs were found to form highly symmetric octamers, both in solution^3^,^23^,^24^ and in the crystalline state^18^,^23^. In the octamers, the contacts between subunits are established between amino acids within the C-terminal domain^18^,^23^,^24^.

In compliance with their central role in metabolism, eukaryotic UGPs are subject to elaborate regulation, including redox mechanisms^25^, phosphorylation^16^ and enzyme sequestration by transient oligomerization^20^. Recently, we reported the monomeric *Leishmania major* UGP (LmUGP) to be regulated by a complex intramolecular mechanism that facilitates large-scale conformational changes^26^.

In the fungal and animal kingdom, the complexity of UGP function is further increased by their octameric organization, which was shown for human UGP (hUGP) to be essential for enzymatic activity^24^. Two crystal structures of octameric UGPs have been obtained to date, the *S. cerevisiae* enzyme^18^ and the shorter isoform 2 of hUGP^23^. Both structures contain no substrates or products and thus represent the apo forms of the enzymes, which share 55% sequence identity^18^ and show close structural homology (Fig. S1e). However, it is unknown how the quaternary structure affects the catalysis and regulation of these enzymes. Revealing such correlations is pivotal to understanding the nucleotide sugar metabolism and may moreover establish a novel basis for the development of drugs that specifically target UGPs of pathogens. On this background, we focused our study at delineating structure-function relationships in hUGP, using protein crystallography in combination with mutational, kinetic and thermostability studies. We thereby resolved the UDP-Glc complexed conformation of the full-length hUGP isoform 1, which represents the first product-bound crystal structure of an octameric UGP, and thus enabled us to identify the binding mode of UDP-Glc and to discover an intermolecular mechanism - we termed it interlock - that stabilizes the sugar-binding region. Importantly, our analysis of the crystal structures of other oligomeric nucleotidyltransferases (NTs) indicated the interlock to be a common feature of these enzymes. In addition, our study evidences that the highly symmetric octameric structure of hUGP increases protein stability and facilitates mild cooperativity in the reverse reaction. The identified properties enable hUGP to meet the extraordinarily complex demands that are placed on an enzyme at the intersection between anabolic and catabolic pathways.

## Results

### Crystal structure of the hUGP1·UDP-Glc complex

Two isoforms - hUGP1 and hUGP2 - are generated from a single gene by alternative splicing (UniProt # Q16851). The two proteins differ by an N-terminal extension of 11 amino acids in hUGP1, the isoform crystallized in this study. The structure of the hUGP1·UDP-Glc complex was solved by molecular replacement using the coordinates of *S. cerevisiae* UGP (PDB ID: 2I5K) as a search model (see Supplementary Methods and Table 1). The asymmetric unit of the crystal contained four hUGP1 molecules (chains A, B, C and D) which formed a non-crystallographic cyclic *C4* tetramer. Monomers within the tetramer contact each other in a heterologous “face-to-back” fashion^27^. In the unit cell, two cyclic *C4* tetramers are stacked onto each other and related by twofold symmetry axes creating the octamer with dihedral symmetry *D4* (Fig. 2a), similar to the one described for *S. cerevisiae* UGP^18^ and the apo-form of hUGP2^23^ (PDB ID: 3R2W). The peptide backbone could be traced throughout most of the protein, except for the N-terminal StrepII-tag, the 23 N-terminal residues of each chain, and residues L359 to L363 of the flexible loop β11–β12 in chain D. The model has an overall good stereochemistry and low coordinate error (Table 1).

### Structure of the hUGP1 subunit and active site in product-bound state

The hUGP1 subunit can be divided into three domains (Fig. 2a, b, c), similar to the subunits of previously solved UGP structures^17^,^18^,^19^,^22^,^23^. The bound UDP-Glc enabled the detailed analysis of the active site (Fig. S1a), which is located in a deep groove at the center of the catalytic domain (Fig. 2b, purple) and consists of a twisted eight-stranded β-sheet (Fig. 2c, white arrows) flanked by α-helices, a structural motif characteristic for nucleotide binding domains. The deepest part of the groove, stretching along the edge of the central β-sheet (Fig. 2b), contains residues L113, N251, and N328 (Fig. S1a) which are strictly conserved in eukaryotic UGPs (Fig. 2c and S2). The nucleotide-binding (NB) loop, the sugar-binding (SB) loop and the eight-stranded β-sheet contribute to the formation of an extensive surface within the groove (Fig. 2b, c). Electron density analysis (Fig. S1a) revealed the binding mode of the product UDP-Glc in the active site of hUGP1, where it is coordinated by the NB-loop, parts of the eight-stranded β-sheet and other residues of the catalytic domain (Fig. 2b, 3d and S1a, b).

To reveal functionally important differences between the product bound state and the apo-state of hUGP, the subunits of the hUGP1·UDP-Glc complex structure (this work, PDB ID: 4R7P) and the hUGP2 apo-structure^23^ (PDB ID: 3R2W) were compared. The respective coordinate errors of 0.4 Å and 0.5 Å for these structures were taken into account when analyzing structural differences. Movements of the N- and C-terminal domains toward the active site upon UDP-Glc binding (closing of the structure) resulted in a slightly more compact geometry with a deeper active site cleft. The bound product caused a mild torsional deformation of the central β-sheet (Fig. 3a and S3a) and moderate conformational changes in the NB-loop (Fig. 3b and S3b). A more prominent rearrangement was observed in the SB-loop region, which undergoes a considerable tilt (Fig. 3c and S3c). Binding of the sugar moiety is aided by the highly conserved SB-loop (Fig. 3c) which consists of residues T286–G293 (Fig. 2c). The phosphate groups of UDP-Glc are coordinated by the active site residues K127, H223 and N251 (Fig. 3d and Fig. S1a, b). Compared to the apo-structure, the side chain of H223 is rotated by 90° in the UDP-Glc state and thus reaches a position which allows formation of an H-bond with the β-phosphate moiety (Fig. 3d and S3d).

With the hUGP1·UDP-Glc complex, we describe the binding mode of UDP-Glc in a UGP from the animal kingdom for the first time.

### Mutational analysis of active site residues

To study the impact of the identified active site residues on hUGP activity, we mutated ten residues, all strictly conserved in eukaryotic UGPs (Fig. S2) and either identified to be involved in product binding (this work) or previously reported to be essential for UGP function^8^,^17^ (Table 2). Amino acids were replaced as reported previously, or by alanine, leucine or glycine, depending on the size of the original residue. Purification attempts after expression in *E. coli* BL21(DE3) demonstrated that the mutants L113G, G222A, H223L and N328L (Table 2) could not be obtained as soluble proteins. All other mutants could be obtained at purities similar to or slightly higher than the wild-type (wt) hUGP1 (Fig. S1c). Enzymatic activities of the successfully purified hUGP1 mutants were determined in the forward reaction and are given relative to the wt hUGP1 activity, defined as 100% (Table 2). All mutants showed a dramatically reduced activity, confirming that the mutated positions are of crucial relevance for hUGP1 activity.

### A quaternary mechanism enables hUGP activity

In the *D4* octameric structure of the hUGP1·UDP-Glc complex (Fig. 2a), all subunits have the same overall conformation. Thus, the transition of the hUGP octamer between the apo- and the product-bound forms is in agreement with the Monod-Wyman-Changeux symmetry model.

Minor differences were observed only in the flexible regions, including the N- and C-termini and the flexible loops α1-α2, α2-α3, α4-α5, β4-α10 and β11-β12 (Fig. 2c). The hUGP1 subunits interact with each other by H-bonds and hydrophobic contacts. The formation of end-to-end connected dimers results from contacts between the C-terminal β23-strands (Fig. 2c) of two opposing subunits. In the octamer, four of these dimers are cross-connected (Fig. 4a) via side-by-side contacts between β16, β17, β20 and β23 (Fig. 2c).

Besides these interactions, previously observed in hUGP2^23^ and *S. cerevisiae* UGP^18^, additional contacts between functionally important areas of neighboring subunits were identified in the hUGP1·UDP-Glc complex. As shown in Fig. 4c, R287 of the SB-loop of one subunit (A′, light green) forms H-bonds with D456 at the base of the C-terminal β-helix domain of the neighboring subunit (A, dark purple), to which it is connected side-by-side (Fig. 4a, b); likewise, R287 (A) interacts with D456 (A′). R287 is part of the SB-loop region (Fig. 3c), which consists of the residues V282-F327 and encompasses the SB-loop, strands β7-β8 and helices α15-α16 (Fig. 3c). This region undergoes a significant conformational change upon UDP-Glc binding (Fig. 3c). Stereochemical analysis of our structural data revealed a number of close intramolecular van-der-Waals contacts within the SB-loop region, indicating conformational strain within the subunit. Energy minimization of a single hUGP1 subunit in the active state with omitted product (see Supplementary Methods) led to conformational relaxation of the hUGP geometry and rendered it more similar to the apo-state (Fig. 3e).

In addition, intermolecular contacts within a side-by-side connected dimer are closer in the product-bound state. As a result, the side-by-side dimer in the active state of hUGP is 6.3% more compact than in the apo-form (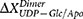 = −2 Å, 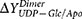 = −4 Å, 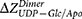 = 0 Å). In particular, the contact between R287 of one protomer and D456 of another protomer becomes considerably stronger in the product-bound state with extra hydrogen bonds formed between their side-chains. The energy minimization of the separate side-by-side dimer from the hUGP1·UDP-Glc complex with omitted product renders the dimer geometry closer to the apo-state.

At the level of the biologically active octamer, the substrate-induced compacting of hUGP (Fig. S1f) becomes even more pronounced. In the active state, the volume of the octamer decreases by 14% (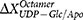 = −8 Å, 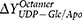 = −3 Å, 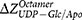 = −11 Å). Energy minimization of hUGP coordinates in the product-bound state applied to the whole octamer, with omitted UDP-Glc, led to a conformational relaxation of the octamer, increase of its volume, and the final geometry close to the apo-state of the enzyme.

Taken together, these analyses indicate that substrate/product binding plays an important role in stabilizing the active state octamer, and that the H-bonds formed between R287 and D456 of neighboring subunits in the octamer are of major importance for stabilization of the sugar-binding site and the SB-loop region. Due to the intermolecular nature of this stabilizing effect, we named it interlock mechanism. In addition, the results of the Molecular Mechanics simulations suggest that the octameric protein matrix of hUGP may contribute to the stabilization of the interlock contact.

Because the residues R287 and D456 involved in the interlock mechanism are only conserved in octameric UGPs from yeast and animals, we next tested whether replacement by amino acids occupying the respective positions in monomeric plant and protozoan UGPs (Fig. 4d) would preserve the activity of hUGP1. The single point mutants R287E, R287L, D456K and the double mutants R287E/D456K and R287L/D456K were constructed and the recombinant soluble proteins produced with purity comparable to or higher than that of the wt enzyme (Fig. 4e). However, activity in the forward and reverse reaction (Fig. 4f) was drastically reduced for all mutants (≤8% of wt), confirming the important role of these positions. To exclude an influence of the introduced mutations on octamerization that would indirectly affect hUGP activity, the oligomerization states were determined by size exclusion chromatography. All mutants migrated like wt hUGP1 (data not shown), allowing us to conclude that the proteins attained native quaternary organization.

Having confirmed that the intermolecular contact between R287 and D456 - the interlock - is important for enzymatic activity, a first molecular interpretation for the functional necessity of hUGP oligomerization was obtained.

### The interlock mechanism exists in other nucleotidyltransferases

The interlock mechanism identified in hUGP caused us to interrogate whether similar mechanisms for stabilization of the sugar-binding region exist in other NTs. We previously demonstrated that in the monomeric LmUGP, stabilization of the sugar-binding region is achieved in an intramolecular fashion (Fig. 4g) termed the lock mechanism^26^. Substrate binding is accompanied by large-scale conformational changes which bring the interacting regions into proximity. Because LmUGP comprises all structural elements required for UGP activity in a single protein chain, we used its product-bound structure^17^ (PDB ID: 2OEG) as a reference to analyze the crystal structures of other oligomeric NTs (listed in Table S2) for the existence of a lock or interlock mechanism. These NTs all use Glc-1-P, but different nucleotide triphosphates as substrate. Comparison of the oligomeric NTs (including hUGP) with monomeric LmUGP revealed the elements involved in nucleoside binding to be conserved in all structures. Likewise, three phosphate-coordinating residues (K127, H223, and K396 in hUGP1) are either conserved or replaced by functional homologs in all analyzed enzymes. In contrast, interlock or lock elements stabilizing the sugar moiety were found to be non-conserved. Analysis of the enzymes' quaternary structures with regard to their molecular mechanisms revealed the conformational changes upon substrate binding to be significantly smaller in the oligomeric NTs than in the monomeric LmUGP. At the same time, a functional intermolecular contact stabilizing the sugar-binding region of a neighboring subunit was discovered in all oligomeric NTs. The particular mode of stabilization varies, but the functional role of the interaction remains the same. For instance, in the octameric uridylyltransferase from *S. cerevisiae*^18^, the contact between subunits stabilizes the SB-loop and an adjacent loop. This contact is mainly established by R276 and D447, corresponding to the hUGP1 interlock residues R287 and D456 (Fig. 4d). In the hexameric *Salmonella typhi* cytidylyltransferase^28^, the part of the active site corresponding to the LmUGP lock region (Fig. 4g) is formed by a neighboring subunit (Fig. 4h). Finally, in the tetrameric thymidylyltransferase from *Pseudomonas aeruginosa*^29^, residues R245-G247 of each subunit interact with residues of the neighboring subunit that form the glucose binding pocket, including the region corresponding to the SB-loop, and help stabilize its structure (Fig. 4i).

Taken together, all analyzed oligomeric NTs feature an interlock mechanism for the stabilization of the sugar-binding region.

### Stability and cooperativity of octameric hUGP1

A multitude of studies showed oligomerization to promote protein stability^30^,^31^,^32^,^33^,^34^,^35^. Asking whether octamerization also influences the stability of hUGP, we applied thermostability measurements and compared wt hUGP1 with two mutants (I498D and Δ501–508), previously shown for hUGP2 to cause dissociation into tetramers and dimers, respectively^24^. While tetramerization of mutant hUGP1 I498D can either be achieved via end-to-end linkage of two side-by-side connected dimers (compare Fig. 4a, e.g. A-A′-C-D′) or vice versa (e.g. A-D′-C-B′), the truncation construct Δ501–508 lacks the terminal β-strand 23 required for the end-to-end contact and therefore most probably consists of two side-by-side connected subunits (e.g. A-A′). Importantly, a single point of inflection was observed for all melting curves (Fig. 5a), indicating that oligomer dissociation and protein denaturation occur concomitantly. Not unexpectedly, the melting temperatures (T_m_) of hUGP1 decreased with the state of oligomerization (Fig. 5a). For the octameric wt, a T_m_ of 55.65 ± 0.05°C was determined while the T_m_ of the tetrameric (I498D) and dimeric (Δ501–508) mutants was decreased to 47.49 ± 0.11°C and 39.44 ± 0.06°C, respectively. The interlock mutants (Fig. 4e, f) were included in this experiment because they retain the native octameric state, although a natural stabilizing interaction (R287/D456, Fig. 4c) was intentionally disturbed. In line with our expectations, the T_m_ of all interlock mutants was only mildly decreased compared to wt hUGP1. For mutant R287L, exemplarily shown in Fig. 5a, a T_m_ of 52.69 ± 0.09°C was measured. These results clearly identified the increased protein thermostability as a second essential function of hUGP octamerization.

Finally, we interrogated whether the recombinant enzyme displays identical kinetic properties as described earlier for hUGP isolated from the natural source^36^. Substrate-velocity-curves were generated for all substrates. In line with the published data, Michaelis-Menten-kinetics were observed for UTP and Glc-1-P in the forward reaction (data not shown). In contrast, the sigmoidal curve obtained for pyrophosphate (PP_i_) in the reverse reaction (Fig. 5b) confirmed a positive cooperative behavior. The Hill-coefficient was 1.7 ± 0.1 and thus in good agreement with the value (1.5–2.5) described for the endogenous enzyme^36^.

The thermostability and kinetic studies provide evidence that the *D4* symmetry of the octameric hUGP extends the functionality of this essential enzyme by improving protein stability and by enabling cooperativity towards PP_i_.

## Discussion

**Figure 1:**
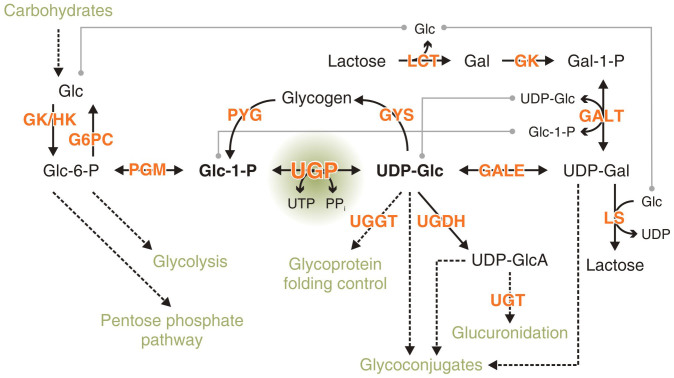
Schematic representation of glucose (Glc) metabolism and the central position of UDP-glucose (UDP-Glc) in cellular pathways. UDP-glucose pyrophosphorylase (UGP, highlighted by green sphere) catalyzes the interconversion of glucose-1-phosphate (Glc-1-P) and uridine triphosphate (UTP) to inorganic pyrophosphate (PP_i_) and UDP-glucose (UDP-Glc). GK/HK: gluco-/hexokinase, G6PC: glucose-6-phosphatase, PGM: phosphoglucomutase, GYS: glycogen synthase, PYG: glycogen phosphorylase, UGGT: UDP-glucose:glycoprotein transferase, UGDH: UDP-glucose 6-dehydrogenase, UGT: UDP-glucuronyltransferase, GALE: UDP-glucose-4-epimerase, GALT: galactose-1-phosphat-uridylyltransferase, GK: galactokinase, LS: lactose synthase, LCT: lactase, Glc-6-P: glucose-6-phosphate, UDP-GlcA: UDP-glucuronic acid, Gal: galactose, Gal-1-P: galactose-1-phosphate, UDP-Gal: UDP-galactose.

**Figure 2:**
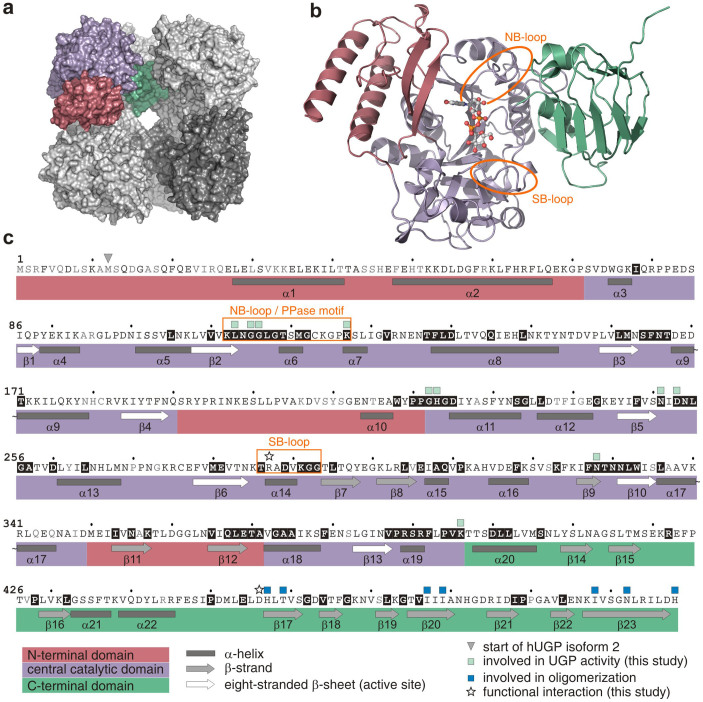
Quaternary, tertiary, secondary and primary structure of hUGP1. (a) Surface representation of octameric hUGP1, with one subunit highlighted in color. The N-terminal, central catalytic and C-terminal domains are shown in red, purple and green, respectively. (b) Ribbon representation of the hUGP1 monomer with bound UDP-Glc shown in ball-and-stick representation. (c) Primary, secondary and tertiary structure elements of hUGP1. Grey, black, and white letters boxed in black indicate non-conserved, partially conserved and strictly conserved residues, respectively, as deduced from a multiple sequence alignment^57^ of hUGP1 with UGP from mouse, chicken, zebrafish, *S. cerevisiae*, *A. thaliana*, barley and *L. major*. See Fig. S2 for full alignment.

**Figure 3:**
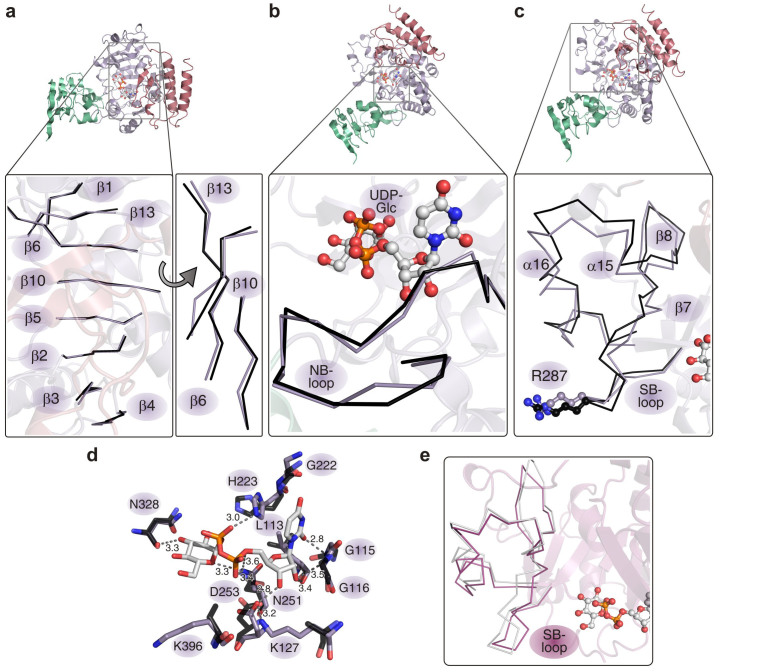
Superposition of hUGP active site elements in apo- (PDB ID: 2R2W; black) and UDP-Glc bound states (this study; purple). (a) The eight-stranded β-sheet, forming the active site cleft (left panel) undergoes a torsional deformation (right panel) upon UDP-Glc binding with maximum displacement of 1.4 Å for the C_α_ atoms and 3.4 Å for the side chains. (b) Coordination of UDP-Glc (ball-and-stick representation) by the NB-loop. The maximum displacement for the NB-loop C_α_ atoms is 1.4 Å and 2.5 Å for the side chains. (c) Conformational rearrangements in the SB-loop region upon UDP-Glc binding. The apo- and UDP-Glc bound conformations are shown in black and purple, respectively. Residue R287, participating in the interlock mechanism, is highlighted in ball-and-stick conformation. The maximum displacement for the SB-loop C_α_ atoms is 3.3 Å and 12 Å for the side chains. (d) Overlay of apo-hUGP2 (semi-transparent, black bonds) and UDP-Glc bound hUGP1 (purple bonds) active site residues involved in product coordination. Numbers correspond to hUGP1 positions. See also Fig. S3 for stereo views of (a)–(d). (e) Conformation of the SB-loop region, comprising residues V282-F327, as observed in the octameric hUGP1·UDP-Glc complex (magenta) and after energy minimization of a single hUGP1 subunit (grey) (see Supplementary Methods).

Positioned at the intersection of anabolic and catabolic pathways, UGP fulfils a vital function in metabolism (shown for animal cells in Fig. 1). Crystal structures of eukaryotic UGPs revealed a high conservation of the active site architecture, but diverse quaternary organizations^17^,^18^,^19^,^22^,^23^. While UGP dimers in plants were shown to represent inactive sequestered forms of the monomeric active species^20^,^37^, hUGP was unequivocally demonstrated to be a functional octamer^24^. The current study aimed at understanding how octamerization is related to hUGP function on a molecular level. Towards this goal we solved the crystal structure of hUGP1 in complex with its product UDP-Glc. When compared to the apo-structure of hUGP2^23^, novel functional interactions between neighboring subunits were discovered and shown to be important for hUGP activity. Moreover, site-directed mutations helped defining the active site pocket, and kinetic and physicochemical measurements demonstrated that octamerization may be needed to fine-tune the metabolic flux of hUGP reactants and to furnish this essential enzyme with the stability required under physiological conditions.

**Figure 4:**
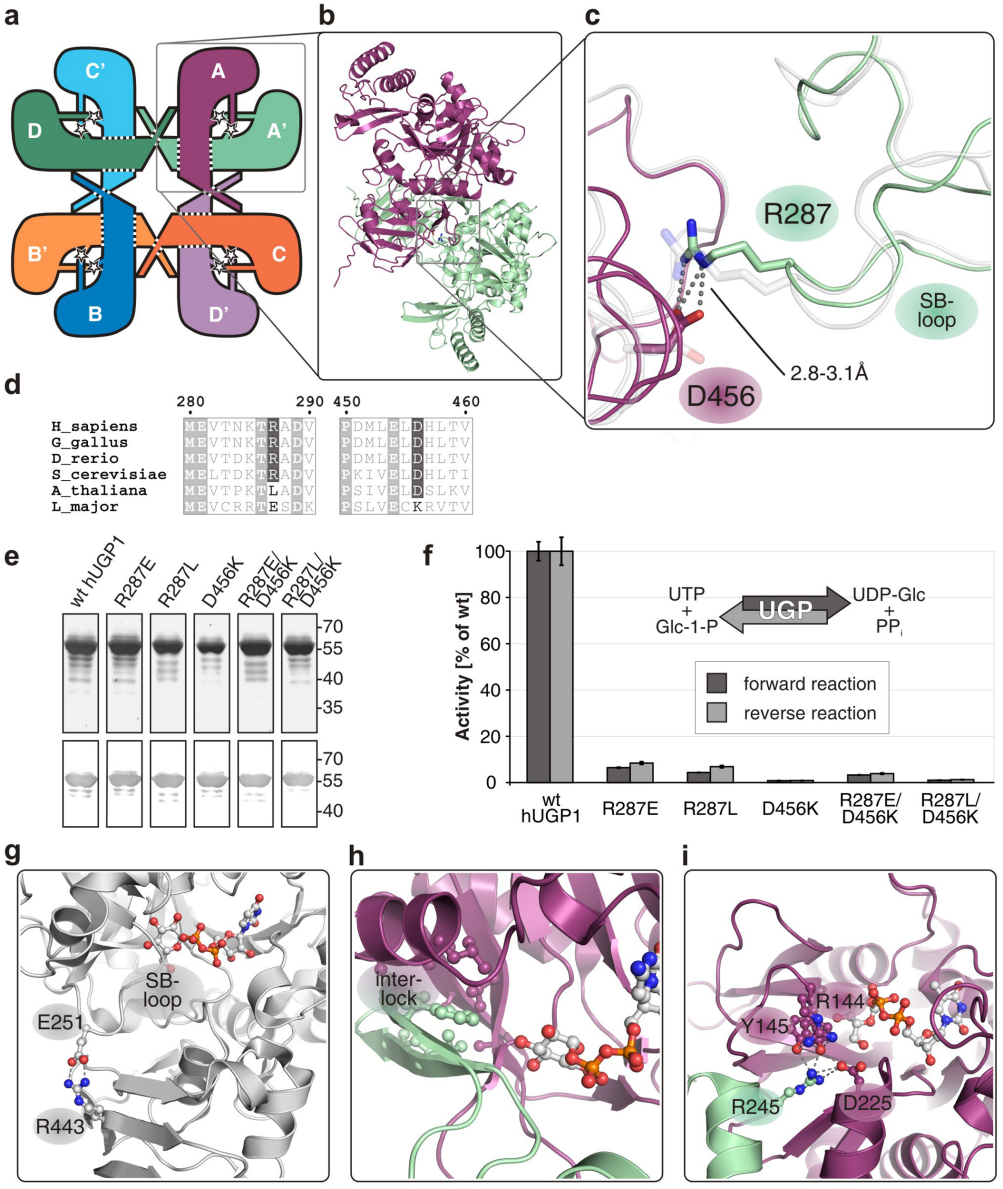
Functional interactions in hUGP and other glucose-activating nucleotidyltransferases. (a) Schematic representation of the hUGP1 octamer, illustrating oligomer-forming contacts (dotted lines) and functional interactions identified in this study (stars). (b) Side-by-side interactions between two hUGP1 subunits. (c) Functional interaction between R287 (green chain) and D456 (purple chain) of hUGP1 by H-bonds (dashed lines), termed interlock. The corresponding region of superimposed apo-hUGP2 is shown in semi-transparent grey. (d) Sections of the hUGP1 sequence containing residues R287 and D456 (boxed in dark grey) in a multiple sequence alignment of animal, fungal, plant and protozoan UGPs, created with MultAlin and ESPript^57^,^58^. Numbering refers to hUGP1. (e) Purified recombinant StrepII-tagged wt hUGP1 and single or double mutants of R287 and D456, analyzed by silver stained SDS-PAGE (top) and Western Blot detecting the StrepII-tag (bottom). (f) *In vitro* activities of wt and mutant hUGP1 in the forward and reverse reaction. Activitities represent means of quadruplicates ± s.d. and are displayed relative to wt hUGP1 activity. (g) Intramolecular lock mechanism stabilizing the SB-loop in monomeric *Leishmania major* UGP (PDB ID: 2OEG). Interacting residues and the bound product are highlighted in ball-and-stick representation (see also (h) and (i)). (h) Interlock stabilizing the sugar-binding region in hexameric *Salmonella typhi* Glc-1-P cytidylyltransferase (PDB ID: 1TZF) by an extended hydrophobic core. (i) Interlock stabilizing the sugar-binding region in tetrameric *Pseudomonas aeruginosa* Glc-1-P thymidylyltransferase (PDB ID: 1G1L).

**Figure 5:**
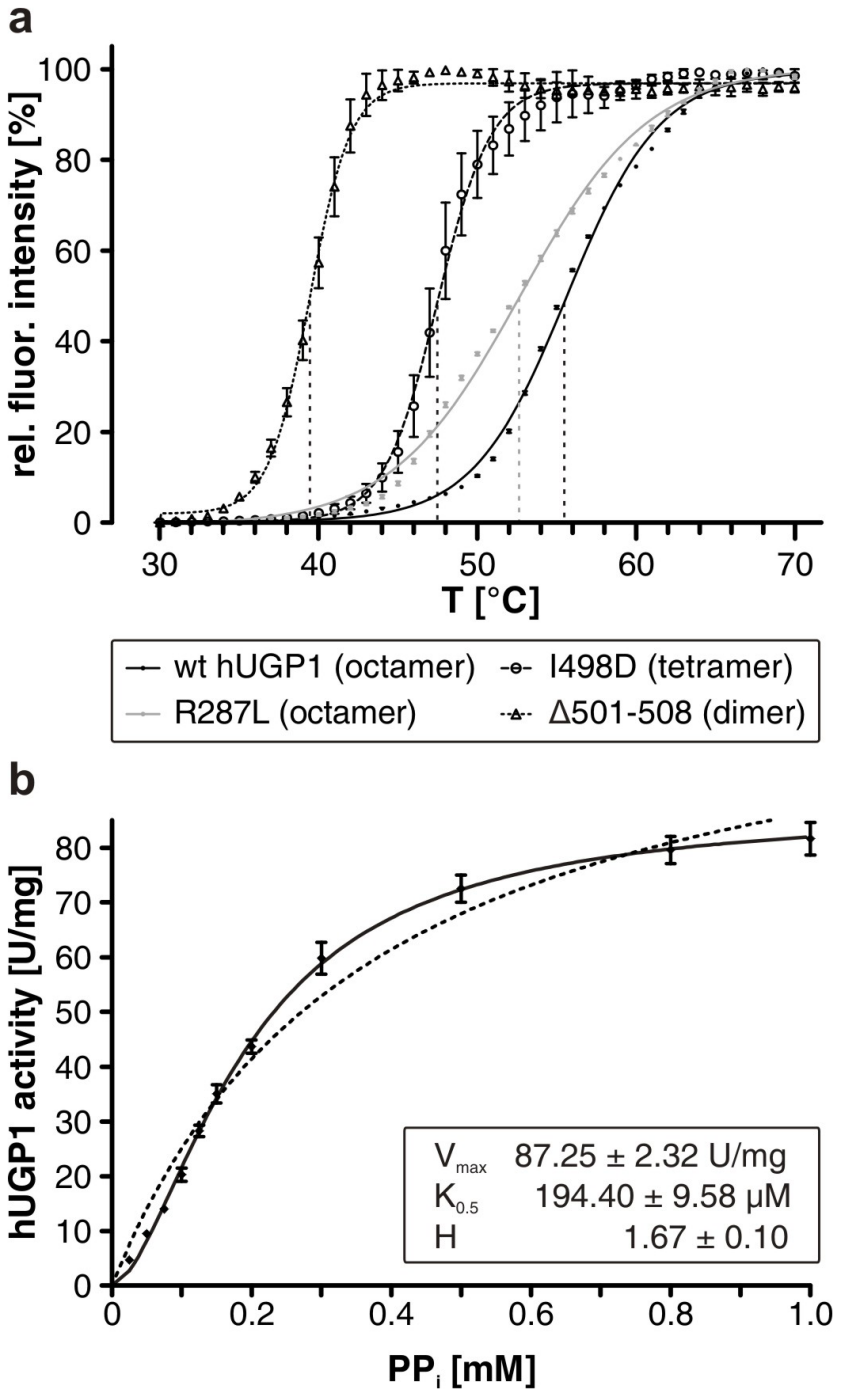
Thermostability and cooperativity of hUGP. (a) Normalized temperature-dependent fluorescence (depicted as relative fluorescence intensity, y-axis) of the octameric wt hUGP1 and nearly inactive mutants displaying octameric (R287L), tetrameric (I498D*) and dimeric (Δ501-508*) quaternary structures. Inflection points of the curves, representing the melting temperature of the proteins, are indicated by dashed vertical lines. Data points represent means of triplicates ± s.e.m. *Oligomerization status of the corresponding hUGP2 mutants determined in solution^24^. (b) *In vitro* activity of wt hUGP1 in the reverse reaction in dependence of PP_i_ concentration. Specific activity (y-axis) is depicted in dependence of substrate concentration (x-axis). Data points represent means of six replicates ± s.e.m. The solid line represents curve fitting to the function y = V_max_ · x^H^/(K^H^ + x^H^). Kinetic parameters V_max_, K_0.5_ and H are given as inset. The dashed black line represents curve fit using the Michaelis-Menten-equation y = V_max_ · x/(K_m_ + x).

Recently we resolved the entire catalytic cycle of UGP from the protozoan parasite *Leishmania major*^26^. In the monomeric LmUGP, UTP binding induces a shift of the NB-loop towards the phosphate moiety and a torsional deformation of the central β-sheet of the catalytic domain, which leads to the formation of the binding site for the glucose ring, a deepening of the active site cleft and movements of the N-terminal/catalytic and C-terminal domains towards each other. The structural rearrangements occurring in hUGP upon UDP-Glc binding qualitatively resemble those observed in LmUGP^17^,^26^, but with smaller amplitude. The limited conformational flexibility of individual subunits in the octameric hUGP, which was similarly observed in other oligomeric NTs^18^,^28^,^29^, is most likely imposed by the enzymes' quaternary organization. To overcome this limitation, which prohibits the stabilization of the sugar-binding region via an intramolecular lock mechanism as in LmUGP, hUGP facilitates an intermolecular mechanism, the interlock. This mutual interaction is established between the SB-loop residue R287 of one subunit and D456 of the neighboring subunit's C-terminus. The two residues are strictly co-conserved in octameric fungal and animal UGPs, but not in monomeric protozoan and plant UGPs (Fig. 4d). Single and double mutations of both residues resulted in dramatic loss of activity, confirming this functional interaction to be of major importance for hUGP activity. Although the interlock involves only two hUGP subunits connected in a side-by-side fashion, our earlier study revealed both dimeric and tetrameric mutants to suffer from a dramatic loss of activity^24^. However, although octamers were shown to exclusively represent the fully active state of hUGP, we also found two C-terminal mutants to have severely impaired activity, despite retaining the octameric state^24^. We therefore hypothesized that not only the octameric state, but also the correct positioning of all hUGP subunits toward each other was crucial for full hUGP activity. In the light of the results obtained in the current study, we hypothesize that the correct establishment of the interlock in all subunits, which is a prerequisite for the formation of the fully active hUGP, can only be achieved in the context of the octameric assembly.

The interlock mechanism, discovered in hUGP and detected in other oligomeric NTs^18^,^28^,^29^ can be considered a quaternary implementation of the lock mechanism in monomeric LmUGP^26^. These findings suggest the lock/interlock mechanism to be a common mechanism for stabilization of the sugar-binding region employed by all sugar-activating enzymes from the NT superfamily, and therefore essential to understanding their molecular mechanisms.

In trypanosomatid parasites such as *Leishmania* and *Trypanosoma*, sugar-activating enzymes - including UGP - represent attractive drug targets since many vital processes within the parasites utilize UDP-Glc and UDP-Gal^38^,^39^,^40^,^41^. However, drugs targeting structurally conserved elements (i.e. the active site; Fig. S1d) of pathogen UGPs would result in cross-reactivity with hUGP and other NTs of the host. Our study revealed the lock and interlock mechanisms of LmUGP and hUGP to utilize non-conserved primary sequence and structural elements, making the lock area in the parasite enzyme an ideal target for the design of selective inhibitors.

Due to the more pronounced conformational flexibility associated with the lock mechanism in LmUGP, UDP-Glc is more tightly enclosed by active site residues than in hUGP (Fig. S1d). These topological differences comply with the observed inability of LmUGP to accommodate UDP-Gal^14^, whereas hUGP can interconvert UDP-Gal and Gal-1-P *in vitro*, albeit with low velocity and substrate affinity^36^,^42^. Notably, this side activity of hUGP is of relevance in individuals suffering from classic galactosemia, a potentially lethal disorder caused by genetic defects in the Gal-1-P uridylyltransferase (GALT, see Fig. 1). Because a minor Gal utilization occurs in these patients despite GALT inactivation^43^, the existence of a separate UDP-Gal pyrophosphorylase was hypothesized^44^,^45^,^46^,^47^. However, because a corresponding enzyme could not be identified, Turnquist *et al*. suggested already in 1974 that UDP-Gal production in galactosemics is a side activity of hUGP^3^. Supporting this hypothesis, Lai and Elsas demonstrated that overexpression of hUGP was able to rescue GALT-deficiency in *S. cerevisiae*^48^.

Since protein oligomerization is often associated with cooperative behavior, increased protein stability and extended regulatory options (reviewed in Marianayagam *et al*.^49^) we asked whether octamerization in hUGP influences these properties. In agreement with kinetic studies on hUGP isolated from natural sources^50^, we observed a mild positive cooperativity for PP_i_ in the recombinant enzyme. The data obtained *in vitro* are, however, not sufficient to interpret the relevance of hUGP cooperativity *in vivo*.

Another functional aspect of the quaternary organization of hUGP1 (and likely all octameric UGPs) was revealed by thermofluorescence studies, demonstrating the octameric arrangement to evoke a significant stability advantage over tetrameric and dimeric hUGP1 mutants. The natural quaternary structure of hUGP may serve to reduce conformational fluctuations and increase functional precision at physiological temperatures. Within the hUGP octamer, the C-terminal domains of two opposing subunits form extended intermolecular β-sheets and a joint, extended hydrophobic core. A similar interaction mode, which increases stability and specificity of the contact, was identified in structures of ribosomal proteins and their complexes^50^,^51^,^52^,^53^, and in proteins involved in DNA replication^54^.

Furthermore, oligomerization of hUGP1 may be advantageous in terms of regulation, because the post-translational modification of one subunit may modify the function of the entire octamer. In *S. cerevisiae*, phosphorylation of UGP at the N-terminus was shown to shift the enzyme's intracellular localization from cytosol to the cell wall. This relocalization entailed a change in the use of UDP-Glc from glycogenesis to cell wall glucan synthesis^16^. Interestingly, phosphorylation of hUGP1 (but not hUGP2) at S11 was detected^55^ and may allow distinct regulation of the two hUGP isoforms, which differ only in the N-terminal sequence. Moreover, in an abundance of phosphoproteomic studies, additional phosphorylation sites including Y186, Y298 and S448 were identified in hUGP (www.phosphosite.org; entry “UGP2 (human)” refers to the gene name and covers both isoforms). Further studies are, however, needed to disclose the function of hUGP phosphorylation *in vivo* and identify the responsible kinases.

In conclusion, oligomerization extends the functionality of hUGP in several ways: it (i) facilitates an intermolecular stabilization of the sugar moiety in the active site (interlock mechanism), (ii) enhances protein stability, (iii) enables mild positive cooperativity observed for the octameric wild-type hUGP1 towards PP_i_ in the reverse reaction, and (iv) may allow regulation of the hUGP octamer by modification of a single subunit. These properties of hUGP help fulfill its functional requirements as a metabolic key enzyme.

## Methods

### Generation of hUGP1 expression construct and point mutations

An expression construct for N-terminally StrepII-tagged hUGP1 (UniProt: Q16851-2) was generated by PCR-amplification of hUGP1 cDNA from plasmid pFL-hUGP1^14^ using primers ACM10 and ACM09 (Table S1). Using the primer-generated BamHI and NotI restriction sites (underlined), the PCR-products were ligated into a modified pET22b expression vector (Novagen) containing an N-terminal StrepII-tag, followed by a thrombin cleavage site (sequence: MASWSHPQFEKGALVPRGS). The resulting plasmid was named pET22b-StrepII-hUGP1. Point mutations were introduced using the overlap extension PCR technique^56^. Briefly, for each point mutation, two internal primers - sense and antisense - were designed which span the mutagenesis site and contain the altered codon for the desired point mutation. Using one flanking and one internal primer each (Table S1), the hUGP1 coding sequence was then amplified from pET22b-StrepII-hUGP1 as two fragments (or three fragments for double mutants), which were able to anneal via the primer-generated overhangs. In a subsequent PCR reaction, the two (or three) fragments for each mutant were annealed to serve as a new template and the full coding sequence including the desired mutation(s) was amplified using the flanking primers ACM10 and ACM09. The resulting PCR products were ligated into the pET22b expression vector in the same way as described above for wild-type (wt) hUGP1. Integrity of all resulting plasmids was confirmed by sequencing (GATC biotech).

### Recombinant expression of StrepII-hUGP1

Ca^2+^-competent *E. coli* BL21(DE3) bacteria were transformed with the respective wt or mutant plasmid by heat shock and grown at 37°C in PowerBroth medium (AthenaES) to an optical density of OD_600 nm_ ≈ 1. Expression of recombinant protein was then induced by supplying the culture with 1 mM IPTG (isopropyl-1-thio-β-D-galactopyranoside). After four hours, the culture was pelleted by centrifugation (6,000 g, 15 min, 4°C), the bacterial pellet washed once with PBS (phosphate-buffered saline) and stored at −20°C until further use.

### Purification of StrepII-tagged wt and mutant hUGP1 by affinity chromatography

For affinity-purification of StrepII-tagged wt or mutant hUGP1, a bacterial pellet harvested from 500 ml of Power Broth expression culture was thawed and resuspended in 25 ml of buffer W (100 mM Tris-HCl pH 8.0, 150 mM NaCl) supplied with protease inhibitors (cOmplete EDTA-free protease inhibitor cocktail, Roche). Bacterial lysis was performed by sonication with a Branson Sonifier (50% duty cycle, output control 5; eight cycles of 30 s alternating with 30 s of rest), while the lysate was being cooled on ice. After removal of cell debris via centrifugation (20,000 g, 15 min, 4°C), the supernatant was passed through a 0.2 μm filter before being loaded onto a 5 ml Strep-Tactin sepharose column (IBA) previously equilibrated with buffer W. Unbound proteins were removed from the column by rinsing with 10 column volumes of buffer W. StrepII-tagged hUGP1 was selectively eluted with a step gradient of 100% buffer E (100 mM Tris-HCl pH 8.0, 150 mM NaCl, 2.5 mM desthiobiotin). The protein-containing fractions were pooled and exchanged to a storage buffer containing 50 mM HEPES pH 7.5, 5 mM MgCl_2_, 1 mM EDTA and 20% sucrose (w/v) using PD-10 desalting columns (Amersham Biosciences), then aliquoted and stored at −80°C after flash-freezing in liquid nitrogen.

### *In vitro* activity assays

In order to comparatively analyze the enzymatic activity of wt and mutant StrepII-hUGP1, the *in vitro* activity was determined in the forward reaction at 25°C in a buffer system composed of 50 mM Tris-HCl pH 7.8 and 10 mM MgCl_2_. The commercially available EnzChek pyrophosphate assay (life technologies), based on detection of PP_i_, was used to continuously monitor the hUGP forward reaction at fixed substrate concentrations of 1 mM UTP and 2 mM Glc-1-P. For the reverse UGP reaction, the formation of UTP was monitored using a continuous enzymatic assay utilizing CTP-synthase as described previously^7^ at fixed substrate concentrations of 1 mM UDP-Glc and 2 mM PP_i_ under the aforementioned buffer conditions. Enzymatic reactions were performed in 100 μl volumes in 96-well half-area flat-bottom microplates (Greiner Bio-One) and initiated by the addition of recombinant hUGP in suitable dilution. Product formation was continuously monitored at 360 nm in a Power-Wave TM 340 microplate reader (Bio-Tek). To calculate the specific activity, protein concentration was determined from the measured absorbance at 280 nm and the proteins' individual extinction coefficient, which was calculated using ProtParam (http://web.expasy.org/protparam/). To determine kinetic parameters, activity was measured as described above for a range of concentrations of one substrate, while the respective other substrate was supplied at constant saturating concentration (see above). The obtained data were plotted and evaluated using GraphPad Prism 4 software. In the forward reaction, data were fitted to the Michaelis-Menten equation y = V_max_ · x/(K_m_ + x). In the reverse reaction with PP_i_ as the varied substrate, due to obvious deviations from Michaelis-Menten-kinetics, the equation y = V_max_ · x^H^/(K^H^ + x^H^) was used for curve fitting to include the possibility of cooperative behavior.

### SDS-PAGE analysis and immunoblotting

To analyze purity and integrity of the purified wt and mutant hUGPs, protein samples were separated according to Laemmli on 10% SDS-polyacrylamide gels overlaid with a 5% stacking gel and proteins were subsequently visualized by silver staining. For Western Blot analysis, proteins were transferred to nitrocellulose membrane (Schleicher & Schüll) and the StrepII-tagged hUGP selectively detected using Strep-Tactin alkaline phosphatase conjugate (IBA).

### Size exclusion chromatography

For determination of the apparent molecular mass of StrepII-tagged hUGP-variants, size exclusion chromatography was performed using a Superdex 200 10/300 GL column (GE healthcare) and a buffer composed of 50 mM HEPES pH 7.5, 5 mM MgCl_2_ and 100 mM NaCl. For protein size calibration, molecular weight marker proteins (Sigma) were subjected to chromatography using the same conditions as for StrepII-hUGP.

### Oligomer stability determination by thermofluorescence

The increase of fluorescence upon temperature dependent unfolding of wt and mutant hUGP1 in presence of the fluorescent dye SYPRO Orange (Sigma; final concentration 5x) was detected while subjecting the proteins to a temperature gradient from 20–90°C. Fluorescence was measured at 20°C between increases to exclude temperature dependent intensities. Data was normalized and a Boltzmann sigmoidal fit was applied (GraphPad Prism 4). The inflection point determines the melting temperature T_m_.

### Crystallization of hUGP isoform 1 in complex with UDP-glucose

Crystals of the recombinant, N-terminally StrepII-tagged hUGP1 were grown at 8°C by vapor diffusion in sitting drop plates. The protein sample contained 10.3 mg/ml of hUGP1, 50 mM HEPES pH 7.5, 5 mM MgCl_2_, 1 mM EDTA, 20% (w/v) sucrose and 4 mM UDP-Glc. 1 μl of the hUGP1·UDP-Glc complex was mixed 1:1 with the reservoir solution containing 100 mM NaCH_3_COO pH 4.8, 520 mM ZnCH_3_COO, 6% (w/v) aminocaproic acid and 75 mM (NH_4_)_2_SO_4_. Prior to flash cooling, the crystals were rinsed in reservoir solution supplemented with 4 mM UDP-Glc and 25% ethylene glycol for cryoprotection.

### Diffraction data collection and structure determination/Molecular modeling and structure analysis

See Supplementary Methods.

## Supplementary Material

Supplementary Information Supplementary Information

## Figures and Tables

**Table 1:** Crystallographic data and refinement statistics for the hUGP1·UDP-Glc complex (PDB ID: 4R7P). Statistics for the highest-resolution shell are shown in parentheses

Data collection	
Beamline	ID14-1, ESRF
Wavelength [Å]	0.9334
Beam size [μm]	30
Temperature [K]	100
Crystal dimensions [μm]	400 × 200 × 70
Data-processing software	XDS, SADABS
Space group	*P3_1_21*
Unit-cell parameters [Å; °]	138.97, 138.97, 311.62; 90, 90, 120
Solvent content *V*_s_ [%]	67.7
Matthews coefficient *V*_M_ [Å^3^ Da^−1^]	3.8
Protein molecules per unit cell	24
Resolution range [Å]	47.62−3.35 (3.47−3.35)
Crystal mosaicity (°)	0.1
Total reflections	630037 (63052)
Unique reflections	50959 (5022)
Multiplicity	12.4 (12.6)
<*I*/*σ*(*I*)>	16.15 (2.36)
Completeness (%)	99.97 (100.0)
* R*_merge_	0.044 (0.427)

Structure refinement	
Model-building software	Coot
Refinement software	PHENIX, REFMAC, CNS
* R_cryst_*	0.1991 (0.2748)
* R*_free_	0.2473 (0.3206)
No. non-hydrogen atoms	15591
No. waters	117
No. protein residues	1935

Geometry	
Coordinate error [Å]	0.4
R.m.s.d., bond lengths [Å]	0.008
R.m.s.d., bond angles [°]	1.10
* B* factor from Wilson plot [Å^2^]	96.3
* B*_norm_(UDP-Glc)*)	1.45
No. of TLS groups	4 (one per chain)

*)*B*_norm_ (UDP-Glc) = *B*_average_ (UDP-Glc) · Occupancy (UDP-Glc)/*B*_average_ (all).

**Table 2:** Active site mutations introduced into hUGP1 and their relative activity in the forward reaction, if available. Asterisks indicate amino acid exchanges guided by literature^8^,^17^. Activities represent means of triplicates ± s.d.

Mutation	Activity [%]
wt hUGP1	100 ± 2.2615
L113G	insoluble
G115D	0.0044 ± 0.0003
G116A*	0.0676 ± 0.0113
K127A*	0.1500 ± 0.0092
G222A	insoluble
H223L*	insoluble
N251L	0.0111 ± 0.0009
D253L	0.0632 ± 0.0043
N328L	insoluble
K396A*	0.0413 ± 0.0028
