# Supplementary material for: A Quaternary Mechanism Enables the Complex Biological Functions of Octameric Human UDP-glucose Pyrophosphorylase, a Key Enzyme in Cell Metabolism
Source: Sci Rep. 2015 Apr 10;5:9618. doi: 10.1038/srep09618 (PMC5381698; doi:10.1038/srep09618)
Supplement: Supplementary Information [file srep09618-s1.pdf]

SUPPLEMENTARY INFORMATION

A Quaternary Mechanism Enables the Complex Biological Functions of Octameric Human UDP-glucose Pyrophosphorylase, a Key Enzyme in Cell Metabolism

Jana Indra Führing[1,§], Johannes Thomas Cramer[1,§], Julia Schneider[1], Petra Baruch[2], Rita Gerardy-Schahn[1,*] and Roman Fedorov[2,3,*]

[1]Institute for Cellular Chemistry, [2]Research Division for Structural Analysis, and [3]Institute for Biophysical Chemistry, Hannover Medical School, Carl-Neuberg-Str. 1, 30625 Hannover, Germany

[§]These authors contributed equally to this study

*Rita Gerardy-Schahn, Gerardy-Schahn.Rita@mh-hannover.de

*Roman Fedorov, Fedorov.Roman@mh-hannover.de

SUPPLEMENTARY METHODS

Diffraction data collection and structure determination - Diffraction data from the crystals of the hUGP1•UDP-Glc complex were collected to 3.35 Å resolution at the ESRF beamline ID14-1 using an ADSC CCD detector (**Table 1**). Data were reduced with XDS[1]. The structure was solved by Molecular Replacement using AMoRe[2] with coordinates of a monomeric subunit of *S. cerevisiae* UGP (PDB ID: 2I5K) as a starting model. During the model refinement, no cut-off for structure-factor amplitudes was used. The Molecular Replacement solution was refined in the torsion-angle simulated annealing procedure of CNS[3,4] followed by energy minimization using deformable elastic network refinement[5,6]. The resulting structure was further refined in multiple rounds of manual real space refinement, using Coot[7] and automated reciprocal space refinement, using PHENIX[8] and REFMAC[9]. The same set of 5% randomly selected R_{free} reflections was used in all refinement protocols. Atomic Displacement Parameters (ADP) were refined with the translation-libration-screw-rotation model (TLS) and individual ADPs. TLS parameters were refined with each macromolecule chain as one TLS-group. Torsion-angle NCS restrictions (restraint σ 2.5°, limit 15.0°) were lifted when R factors converged. Final electron density was calculated using the density modification (DM) procedure[10]. The DM was performed in a solvent flattening mode, using Hendrickson-Lattman coefficients from the refined protein model with omitted substrate and ligands as an initial approximation. The composite $2F_o – F_c$ electron density map was calculated using weighted structure factor and modified phases resulting from the DM procedure. Structure comparisons were performed using SSM and LSQ superposition in Coot[11].

Molecular modeling and structure analysis - Molecular mechanics energy minimization of an individual hUGP monomer was performed using CNS[3,4], using an explicit water model with periodic boundary conditions in an artificial unit cell with *P1* symmetry containing the hUGP monomer and three coordination spheres of water around the protein. The starting geometry was obtained from coordinates of the individual monomer (chain A) of the hUGP1•UDP-Glc complex. Stereochemical structure analysis was performed using Coot[7].

SUPPLEMENTARY RESULTS

Figures

(Next page) Figure S1 Active site analysis of hUGP and species comparisons. **(a)** Portion of the composite $2F_o$–F_c electron density omit map, calculated using weighted structure factor and modified phases resulting from the DM procedure (See Experimental Procedures for details). The map was contoured at 0.8 σ, around UDP-Glc (grey bonds) and surrounding active site residues (purple bonds) shown in stick representation. Red, blue and orange atom colours represent oxygen, nitrogen and phosphorus, respectively. **(b)** LigPlot illustration[12] of the interaction of hUGP1 with UDP-Glc, displaying atoms (black: carbon; blue: nitrogen; red: oxygen; orange: phosphorus) of UDP-Glc (grey bonds) and hUGP1 residues (purple bonds) involved in hydrogen bonds (dotted lines) and hydrophobic contacts (half circles). **(c)** Purified recombinant StrepII-tagged wt hUGP1 and active site mutants, analyzed by silver stained SDS-PAGE (top) and Western Blot detecting the StrepII-tag (bottom). **(d)** Ribbon representation of one subunit of octameric hUGP1 (top, dark grey, this work) and monomeric *L. major* UGP (bottom, light grey, PDB ID: 2OEG). Conserved structural elements are highlighted in color i.e. the NB-loop (green), the central β-sheet (blue) and the SB-loop (orange). UDP-Glc is shown in ball-and-stick representation with oxygen, nitrogen and phosphorus atoms displayed in red, blue and orange, respectively. **(e)** Comparison of the quaternary structure and active site architecture of octameric UGPs from *S. cerevisiae* (PDB ID: 2I5K) and *H. sapiens* (isoform 2, PDB ID: 3R2W) in the apo-form. Top row: surface representation of apo *S. cerevisiae* UGP (green) and hUGP2 (blue) octamers, with individual subunits depicted in different hues. Bottom: active site residues of *S. cerevisiae* UGP (light grey bonds and labels) and hUGP2 (black bonds and labels), corresponding to residues shown to be involved in product binding and activity in this study (compare **Fig. 3d**). **(f)** Schematic representation of the compaction of the hUGP octamer in the UDP-Glc bound form. The outer cuboid frame (golden) represents the space filled by the apo hUGP2 octamer, the inner cuboid encloses the hUGP1*UDP-Glc complex (subunits represented by individual colours). Dimensions for both cuboids in Å are given along the respective outer and inner edges (blue text), differences are given along the X, Y and Z axis (black text).

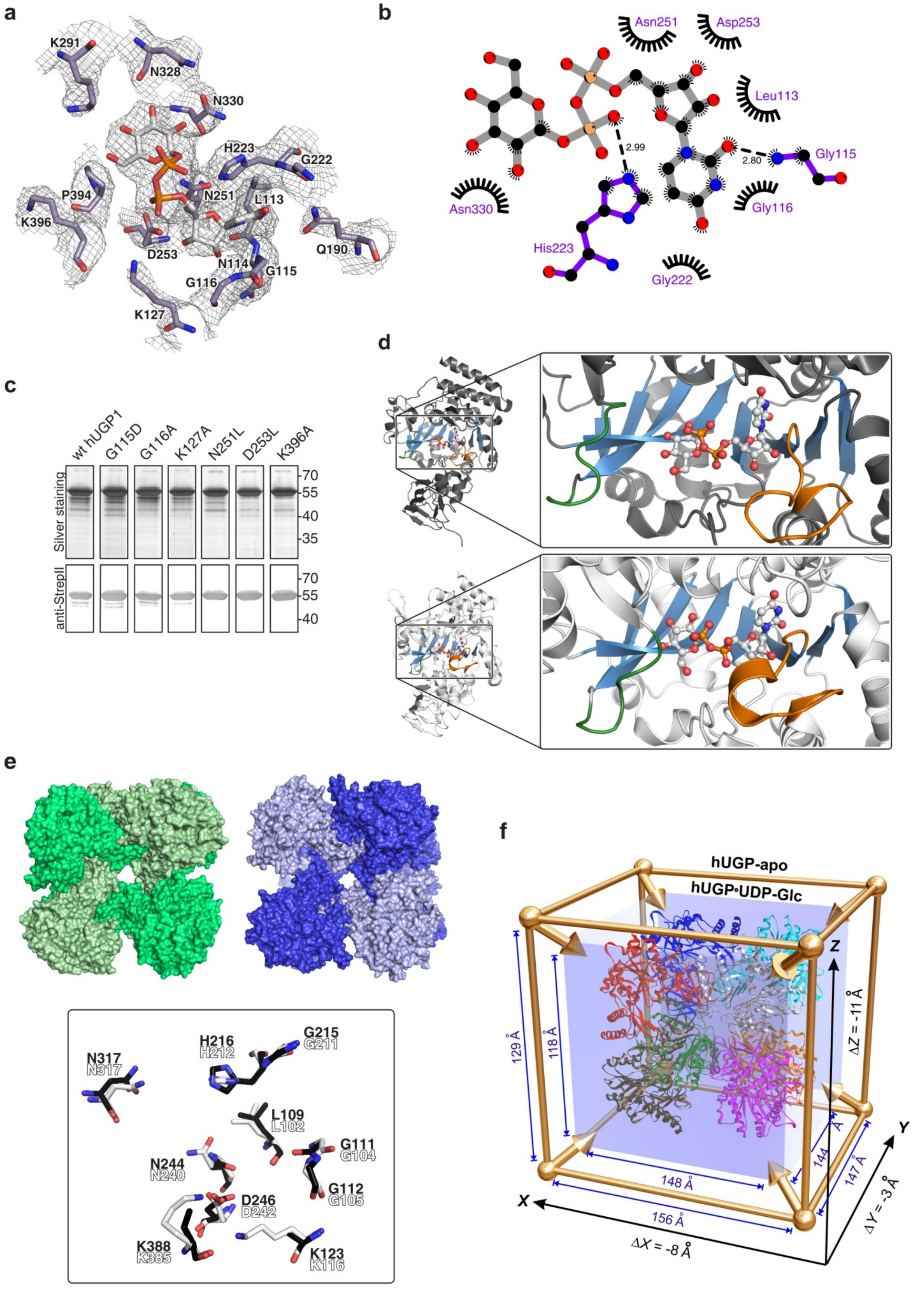
a
K291
N328
N330
H223
G222
P394
N251
L113
K396
D253
N114
Q190
G116
G115
K127
b
Asn251
Asp253
Leu113
2.99
2.80
Gly115
Asn330
Gly116
His223
Gly222
c
wt hUGP1
G115D
G116A
K127A
N251L
D253L
K396A
Silver staining
anti-StrepII
70
55
40
35
70
55
40
d
e
H216
H212
G215
G211
N317
N317
L109
L102
G111
G104
N244
N240
G112
G105
D246
D242
K388
K385
K123
K116
f
hUGP-apo
hUGP•UDP-Glc
129 Å
118 Å
Z
ΔZ = -11 Å
148 Å
156 Å
144 Å
147 Å
X
Y
ΔX = -8 Å
ΔY = -3 Å

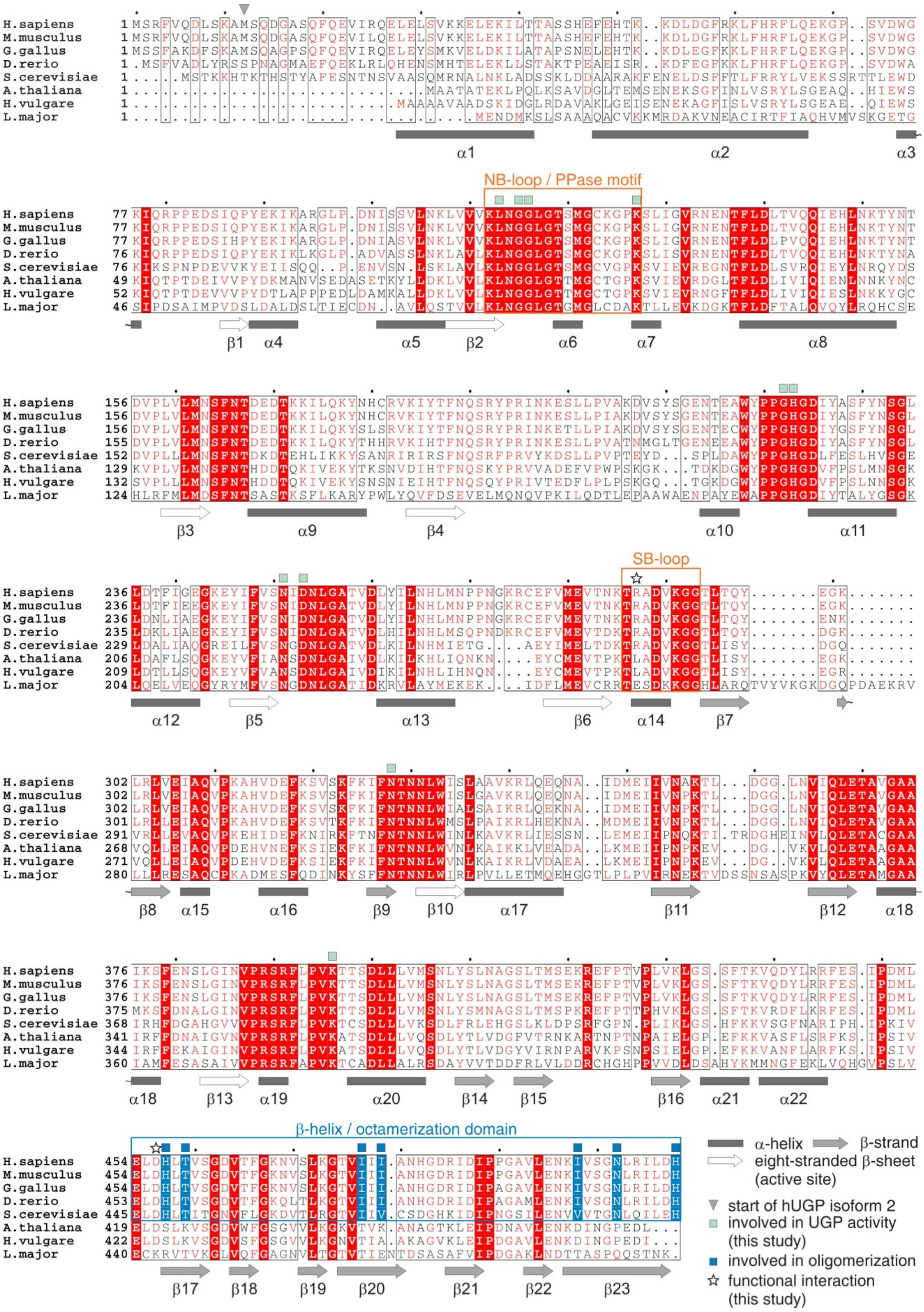
NB-loop / PPase motif
SB-loop
β-helix / octamerization domain
H.sapiens
M.musculus
G.gallus
D.rerio
S.cerevisiae
A.thaliana
H.vulgare
L.major
α-helix
β-strand
eight-stranded β-sheet (active site)
start of hUGP isoform 2
involved in UGP activity (this study)
involved in oligomerization
functional interaction (this study)

(Previous page) Figure S2 Multiple sequence alignment of eukaryotic UGPs from human, mouse, chicken, zebrafish, *S. cerevisiae*, *A. thaliana*, barley and *L. major* created with MultAlin[16] and ESPript[17]. Black, red, and white letters boxed in red indicate non-conserved, partially conserved and strictly conserved residues, respectively. White letters boxed in blue indicate residues that are involved in octamerization and strictly conserved only among animal UGPs. Secondary structure elements, functional loops and domains of hUGP1 and residues mutated in this study are highlighted underneath and above the alignment.

(Next page) Figure S3 Conformational rearrangements of hUGP associated with UDP-Glc binding. **(a) – (d)** Wall-eye stereo views of the **s**uperposition of hUGP active site elements in the apo- (PDB ID: 2R2W; black) and UDP-Glc bound state (this study; purple). **(a)** The eight-stranded β-sheet, forming the active site cleft (left panel) undergoes a torsional deformation (right panel) upon UDP-Glc binding. **(b)** Coordination of UDP-Glc (ball-and-stick representation) by the NB-loop. **(c)** Conformational rearrangements in the SB-loop region upon UDP-Glc binding. The apo- and UDP-Glc bound conformations are shown in black and purple, respectively. Residue R287, participating in the interlock mechanism, is highlighted in ball-and-stick conformation. **(d)** Overlay of apo-hUGP2 (semi-transparent, black bonds) and UDP-Glc bound hUGP1 (purple bonds) active site residues involved in product coordination.

a

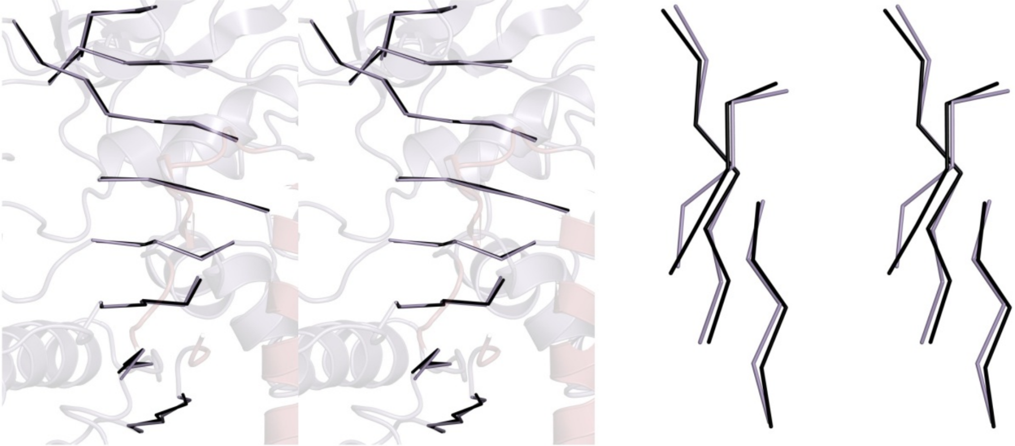

b

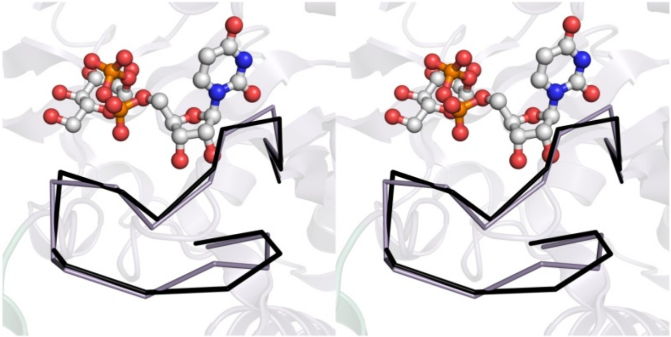

c

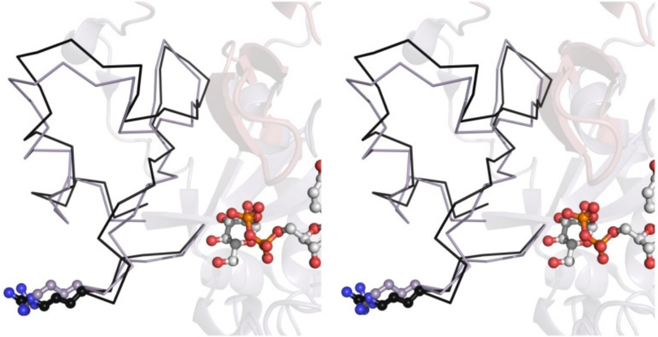

d

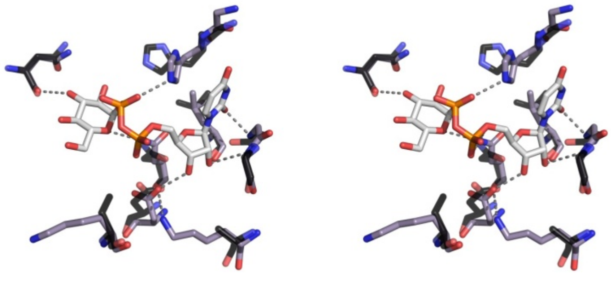

Tables

Table S1 Primers used for the amplification of wt or mutant human UGP1. Each mutant hUGP1 coding sequence was assembled from two (for single mutants) or three (for double mutants) fragments which were generated using the respective mutagenesis primers and hUGP1 primers ACM10 and ACM09 for N- and C-terminal fragments, respectively. Codons that were changed in order to introduce point mutations are given in uppercase. BamHI and NotI restriction sites introduced for ligation of the PCR-products into the expression vector are underlined.

Mutation	Primer names and sequences	
wt	ACM10	cgcgggatcctcgagatttgtacaagatc
	ACM09	atatgcggccgctcagtggtccaagatgc
L113G	JF53	gctggttcccaaaccaccattCCCtttcaccaccactagtttgttc
	JF52	gaacaaactagtggtggtgaaaGGGaatggtggtttgggaaccagc
G115D	JF35	gcccatgctggttcccaaaccATCattgagtttcaccaccactag
	JF34	ctagtggtggtgaaactcaatGATggtttgggaaccagcatgggc
G116A	JF55	gcccatgctggttcccaaTGCaccattgagtttcaccaccactag
	JF54	ctagtggtggtgaaactcaatggtGCAttgggaaccagcatgggc
K127A	JF37	cctcacaccaatcagactTGCagggcctttgcagcccatgc
	JF36	gcatgggctgcaaaggccctGCAagtctgattggtgtgagg
G222A	JF59	ctggcgtaaatatcaccatgTGCtggagggtaccaagcttctg
	JF58	cagaagcttggtaccctccaGCAcatggtgatatttacgccag
H223L	JF39	ctggcgtaaatatcaccCAAacctggagggtaccaagcttctg
	JF38	cagaagcttggtaccctccaggtTTGggtgatatttacgccag
N251L	JF41	ctgtggcacccagattatctatCAAagacacaaaaatatactctttgccttctcc
	JF40	ggagaaggcaaagagtatatttttgtgtctTTGatagataatctgggtgccacag
D253L	JF43	cagatccactgtggcacccagattCAAtatgttagacacaaaaatatactc
	JF42	gagtatatttttgtgtctaacataTTGaatctgggtgccacagtggatctg
N328L	JF67	gctgcaagagaaatccataggttgtttgtCAAaaatattttgaactttgatacagacttgaactcg
	JF66	cgagttcaagtctgtatcaaagttcaaaatatttTTGacaaacaacctatggatttctcttgcagc
K396A	JF47	gcaagagatctgatgtggtTGCgacaggcagaaaacggctcc
	JF46	ggagccgttttctgcctgtcGCAaccacatcagatctcttgc
R287E	JIF88	ggaagtcacaaataaaacaGAGgcagatgtaaagggcg
	JIF89	cgccctttacatctgcCTCtgttttatttgtgacttcc
R287L	JIF90	ggaagtcacaaataaaacaCTTgcagatgtaaagggcg
	JIF91	cgccctttacatctgcAAGtgttttatttgtgacttcc
D456K	JIF92	ccagatatgcttgaattgAAAcacctcacagtttcagg
	JIF93	cctgaaactgtgaggtgTTTcaattcaagcatatctgg

Table S2 Oligomeric nucleotidyltransferases analyzed with regard to the presence of an interlock mechanism.

Enzyme (EC Nr.)	Organism	Oligomeric state	PDB ID (Reference)
Glucose-1-phosphate uridylyltransferase (2.7.7.9)	*Saccharomyces cerevisiae*	Octameric	2I5K[13]
Glucose-1-phosphate cytidylyltransferase (2.7.7.33)	*Salmonella typhi*	Hexameric	1TZF[14]
Glucose-1-phosphate thymidylyltransferase (2.7.7.24)	*Pseudomonas aeruginosa*	Tetrameric	1G1L[15]

References

1. Kabsch, W. XDS. *Acta Crystallogr.D.Biol.Crystallogr.* **66**, 125-132 (2010).
2. Navaza, J. AMoRe: an automated package for molecular replacement. *Acta Crystallogr.A* **A50**, 157-163 (1994).
3. Brunger, A. T. et al. Crystallography & NMR system: A new software suite for macromolecular structure determination. *Acta Crystallogr.D.Biol.Crystallogr.* 54, 905-921 (1998).
4. Brunger, A. T. Version 1.2 of the Crystallography and NMR system. *Nat.Protoc.* 2, 2728-2733 (2007).
5. Schröder, G. F., Brunger, A. T., & Levitt, M. Combining efficient conformational sampling with a deformable elastic network model facilitates structure refinement at low resolution. *Structure* **15**, 1630-1641 (2007).
6. Schröder, G. F. G., Levitt, M., & Brunger, A. T. A. Super-resolution biomolecular crystallography with low-resolution data. *Nature* **464**, 1218-1222 (2010).
7. Emsley, P., Lohkamp, B., Scott, W. G., & Cowtan, K. Features and development of Coot. *Acta Crystallogr.D.Biol.Crystallogr.* **66**, 486-501 (2010).
8. Adams, P. D. *et al.* PHENIX: a comprehensive Python-based system for macromolecular structure solution. *Acta Crystallogr.D.Biol.Crystallogr.* **66**, 213-221 (2010).
9. Murshudov, G. N., Vagin, A. A., & Dodson, E. J. Refinement of macromolecular structures by the maximum-likelihood method. *Acta Crystallogr.D.Biol.Crystallogr.* **53**, 240-255 (1997).
10. Cowtan, K. *Joint CCP4 and ESF-EACBM Newsletter on Protein Crystallography 31*(1994).
11. Krissinel, E. & Henrick, K. Secondary-structure matching (SSM), a new tool for fast protein structure alignment in three dimensions. *Acta Crystallogr.D.Biol.Crystallogr.* **60**, 2256-2268 (2004).
12. Wallace, A. C., Laskowski, R. A., & Thornton, J. M. LIGPLOT: a program to generate schematic diagrams of protein-ligand interactions. *Protein Eng* **8**, 127-134 (1995).
13. Roeben, A. et al. Structural basis for subunit assembly in UDP-glucose pyrophosphorylase from

Saccharomyces cerevisiae. *J.Mol.Biol.* **364**, 551-560 (2006).

14. Koropatkin, N. M., Cleland, W. W., & Holden, H. M. Kinetic and structural analysis of alpha-D-Glucose-1-phosphate cytidylyltransferase from Salmonella typhi. *J.Biol.Chem.* **280**, 10774-10780 (2005).

15. Blankenfeldt, W., Asuncion, M., Lam, J. S., & Naismith, J. H. The structural basis of the catalytic mechanism and regulation of glucose-1-phosphate thymidylyltransferase (RmlA). *EMBO J.* **19**, 6652-6663 (2000).

16. Corpet, F. Multiple sequence alignment with hierarchical clustering. *Nucleic Acids Res.* **16**, 10881-10890 (1988).

17. Robert, X. & Gouet, P. Deciphering key features in protein structures with the new ENDscript server. *Nucleic Acids Res.* **42**, W320-W324 (2014).
